# Effect of positive event recording based on positive psychology on healthy behaviors and readmission rate of patients after PCI: a study protocol for a prospective, randomized controlled trial

**DOI:** 10.1186/s13063-022-06964-9

**Published:** 2022-12-13

**Authors:** Yao-Yao Hu, Xin Jiang, Fang-Ying Mao, Jing Zhang, Lin Liu, Jie Gu, Qing Wu, Chun Li

**Affiliations:** 1 Department of Cardiology, the First Affiliated Hospital of Soochow University, Suzhou, China; 2 Nursing Department, the Affiliated Wuxi People’s Hospital of Nanjing Medical University, Wuxi, China; 3 School of Nursing, Soochow University, Suzhou, China

**Keywords:** Positive, Coronary heart disease, Behaviors, Readmission rate, Randomized controlled trial

## Abstract

**Background:**

Unhealthy behaviors of coronary heart disease (CHD) patients are closely related to the occurrence of major heart events, which increases the readmission rate and brings a heavy economic burden to families and society. Therefore, it is necessary for health care workers to take active preventive and therapeutic measures to keep or establish healthy behaviors of patients. Positive psychological intervention has been proved to be effective, but it has not been reported in the field of CHD in China. The purpose of this study is to explore the effects of positive event recording based on positive psychology on the healthy behaviors, readmission rate, and anxiety of patients with CHD, in order to provide new ideas for the development of secondary prevention strategies for CHD.

**Methods:**

This is a prospective, single-center, randomized controlled trial (RCT). The subjects will be enrolled from the Department of Cardiology, the First Affiliated Hospital of Soochow University. There are 80 cases in total; according to the random number table, the subjects are randomly divided into the intervention group (*n* = 40) and the control group (*n* = 40). The patients in the intervention group will receive the intervention of recording positive events once a week for 3 months, while the patients in the control group receive conventional nursing. The primary outcomes will include healthy behaviors, readmission rate, and anxiety, and the secondary outcomes will include psychological capital, subjective well-being, and corresponding clinical laboratory indicators. The protocol was approved by the Medical Ethics Committee of Soochow University (approval no. SUDA20200604H01) and is performed in strict accordance with the Declaration of Helsinki formulated by the World Medical Association. All participants provide written informed consent.

**Discussion:**

This study will verify whether positive event recording based on positive psychology can make patients maintain healthy behaviors, reduce readmission rate, and improve anxiety after PCI. Then, this study will provide new ideas and references for the development of secondary prevention strategies for patients with CHD.

**Trial registration:**

Chinese Clinical Trials Registry 2000034538. Registered on 10 July 2020.

**Supplementary Information:**

The online version contains supplementary material available at 10.1186/s13063-022-06964-9.

## Background

Coronary heart disease (CHD) is an acute myocardial ischemia and hypoxia disease caused by coronary artery stenosis, which is one of the highest mortality diseases in the world [1]. With the increasing incidence rate and mortality of CHD, it has resulted in a large amount of medical expenses, making coronary heart disease one of the main reasons for the economic burden of chronic non-communicable diseases in China [2, 3]. At present, percutaneous coronary intervention (PCI) has become one of the main methods for treating CHD because of its characteristics such as low trauma, fast recovery, and short hospitalization time. Although PCI significantly reduces the symptoms of CHD, reduces mortality, and significantly improves the quality of life [4], there is still a risk of major adverse cardiovascular events (MACE) in some patients, which can seriously affect prognosis and increase readmission rates [5, 6].

The prognosis of patients with CHD is determined by various factors. Studies have shown that unhealthy behaviors increased the risk of in-stent restenosis (ISR) and the occurrence of MACE [6, 7]. In addition, negative psychology also affected the prognosis of patients after PCI. Iles-Smith et al. [8] confirmed that anxiety was a predictor of readmission. Frasure-Smith et al. [9] concluded that mood disorders were associated with the occurrence of MACE within 2 years in patients with CHD. However, one study has proved that emotional intervention in elderly patients with heart failure can help them improve heart function, reduce the occurrence of falls, and improve the quality of life [10]. It has also been found that psychological interventions for patients can improve treatment cooperation and can also help patients change their lifestyle and establish healthy behaviors [11–13]. Therefore, patients will benefit most if all known risk factors are controlled.

Positive psychology is a part of psychology, which mainly helps people to form good psychological quality and behavior patterns, focusing on positive subjective experience, positive personality traits, and positive social environment, and fully exploiting the individual’s own strength [14, 15]. At present, positive psychology has been widely used in many fields abroad, such as obstetrics and gynecology, and diabetes. Corno et al. [16] found that positive psychological intervention can help pregnant women reduce anxiety and depression and improve happiness index. Huffman et al. [17] carried out positive psychological intervention on patients with type 2 diabetes and found that it can help patients improve treatment compliance; also, Hoeppner et al. [18] showed that positive psychological intervention can help people quit smoking. However, the development of positive psychology in China is relatively less, mainly in the fields of education, mental illness, etc. The study by Lan et al. [19] clarified that psychological intervention based on positive psychology can help patients maintain a good psychological state, improve depression, and improve self-care ability. Zhu et al. [20] also confirmed that positive psychological intervention can improve the disability acceptance and self-care ability of stroke inpatients.

The application of positive psychology is currently done in a number of different ways, such as expressive writing, mindfulness therapy, gratitude therapy, hope therapy, etc. These methods have a positive effect on reducing negative emotions and improving the subjective well-being of patients. Writing is a unique human behavior of information expression, communication, and creation, and the positive event recording in this study is to express the positive experience or feelings related to the individual through writing [21], which has also been applied to relevant fields and achieved certain curative effects. For example, an intervention study of 411 participants, conducted by Seligman [22] showed that recording positive events significantly improved the well-being of those who were involved. Burton and King [23] found that recording positive events can help people improve their cognition. Suhr et al. [24] have shown that the benefits of writing positive events to help people with mental illness stabilize their mental health. Chinese scholar Xu et al. [25] found that writing positive events and expressing can effectively improve human immunodeficiency virus (HIV)/acquired immune deficiency syndrome (AIDS) patients’ self-discrimination and negative emotions and improve the self-control level. However, there are few reports about positive events recorded in patients after PCI in China, and most of the positive psychological intervention programs in China have poor repeatability and lack of systematic and targeted intervention programs. In view of the simple operation of positive event recording, the time is short, economical, and convenient, and the effect is remarkable, it is necessary to carry out positive psychological intervention for patients after PCI in China to explore its intervention effect and formulate a more suitable positive psychological intervention program for patients with CHD in China.

The purpose of this study is to explore whether the implementation of positive event recording has an impact on the healthy behaviors, readmission rate, and anxiety of patients after PCI in the theoretical framework of positive psychology, in order to provide a reference for improving the quality of specialized nursing and formulating secondary prevention strategies for patients with CHD.

### Objectives

The general aim of this study is to evaluate the efficacy of positive event recording in patients after PCI.

The primary objective is to explore whether recording positive events will promote or maintain healthy behaviors, reduce readmission rate, and improve anxiety of patients after PCI.

The secondary objective is to explore whether recording positive events can promote the positive emotions, subjective well-being, and clinical laboratory indicators (such as total cholesterol (TC), triglyceride (TG), high-density lipoprotein (HDL), low-density lipoprotein (LDL), height and weight, etc.) of patients after PCI.

## Methods

### Study design and setting

**Fig. 1:**
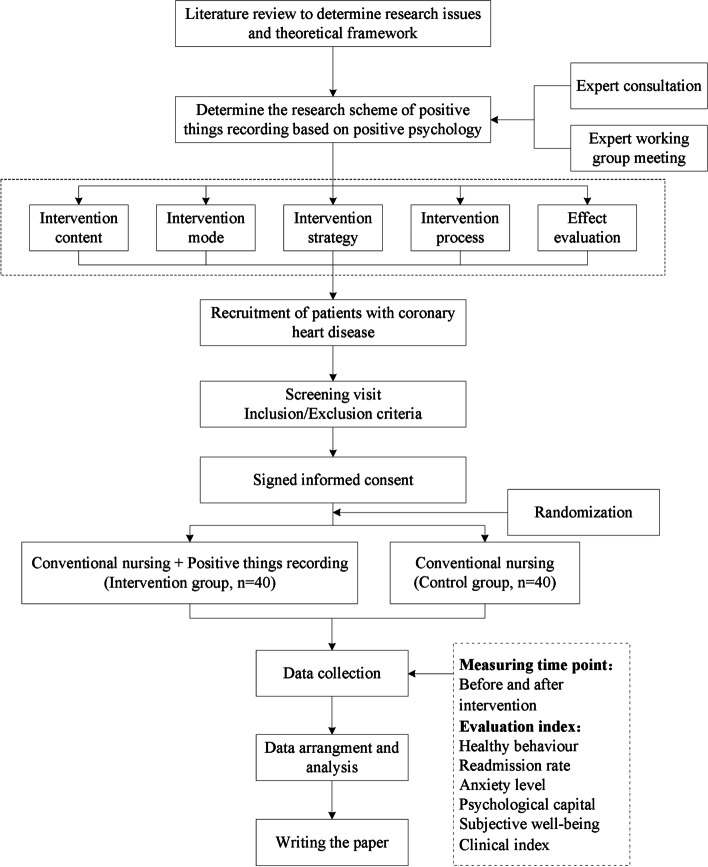
Schematic flow diagram of the study

This is a randomized controlled trial, which will be conducted in the Department of Cardiology, the First Affiliated Hospital of Soochow University. A written informed consent will be obtained from each individual by the researcher after the individual has received a sufficient explanation and period of time in which to make a thoughtful decision. All the patients will be randomized 1:1 into the intervention group (*n* = 40) or the control group (*n* = 40). The intervention group is given 3-month positive event recording intervention on the basis of conventional nursing, while the control group received conventional nursing, and both groups will be followed up for 3 months at the end of the intervention. The Cardiovascular Medicine Department of the First Affiliated Hospital of Soochow University is currently the largest cardiovascular disease diagnosis and treatment center in South Jiangsu Province, China. It has 180 beds, four cardiac catheterization rooms with advanced equipment, and a cardiac supersonic room and cardiac function room. Annually, an average of 5000 open-heart surgery candidates and 400 emergency operations are treated in the department. The protocol design is based on the Consolidated Standards of Reporting Trials (CONSORT) guidelines and Standard Protocol Items: Recommendations for Interventional Trials (SPIRIT) checklist (see Additional file 3). Figure 1 presents a schematic flow diagram of the study.

### Participants

#### Inclusion criteria

Participants must meet the following criteria:

1. Those who are aged 18 years or older
2. Those who meet the diagnostic criteria of coronary heart disease in the ninth edition of Internal Medicine, including stable angina, unstable angina, myocardial infarction, and acute coronary syndrome [26]
3. Those who are after PCI
4. Those who agree to participate and provide written informed consent
5. Those who have been living in Suzhou for a long time or are not leaving Suzhou for half a year
6. Those who are clear-headed, normal-thinking, and able to communicate with each other

#### Exclusion criteria

Applicants who meet any of these conditions cannot participate in this study:

1. Those who have severe cognitive impairment, mental illness, and non-cooperation
2. Those who have other serious diseases that affect survival, such as advanced cancer
3. Those who could not record for 4 consecutive weeks
4. Other reasons determined by the investigators that make participation in the clinical trial inappropriate

#### Withdrawal criteria

Participants who meet the criteria summarized below are withdrawn from the study. The subjects who are withdrawn after randomization will be followed up for outcomes. Reasons for withdrawal will be documented in follow-up records and data will be analyzed using the intention-to-treat (ITT) principle.

1. All subjects have the right to withdraw for any reason
2. Those who could not complete the test as required, with serious adverse events and complications
3. Failed to have three consecutive telephone connections during the follow-up period at the end of the study

### Randomization

#### Sequence generation

Eligible participants who will provide written consent to take part in the clinical trial will be randomly assigned to an intervention group or a control group with an allocation ratio of 1:1. Random sequencing will be generated by an independent professional statistician using the SPSS 25.0 software to generate 80 random numbers. This statistician will not be involved in this study and only help us generate the random numbers and allocation sequence.

#### Allocation concealment

The serial number assigned to each patient will be placed in an opaque sealed envelope in the custody of a person not involved in the specific study to ensure concealment.

#### Implementation

The allocation sequence will be generated by a statistical expert using the SPSS 25 software package. LL will be in charge of enrolling the patients. On the day of the group assignment, the allocation schedule will be issued to the program manager, who will assign subjects to the study group.

### Blinding

Member of the research team who enters the information is the only ones who know about the participants’ allocation. The data collectors, random allocation program makers, data analysts, and outcome evaluators are blinded to group allocation. And the participants are similarly blinded to their own group allocation. Participants are similarly blinded to their own group allocation and they will be told not to share their positive event records with others. Results from the outcome measures will not be revealed to the participants until after all recruitment, treatment, and assessments have been performed for all 80 participants.

### Procedures

#### Recruitment

Participants will be recruited from The First Affiliated Hospital of Soochow University located in Suzhou, China. This institution will advertise the trial by posting recruitment posters on the hospital website or bulletin board.

#### Study schedule

The items to be measured at each visit are listed in Table 1.

**Table 1:** Schedule of enrollment, intervention, and assessment

Items	Screening	Post-allocation							3 months after the trial
	Visit 1 (day − 7 to − 2)	Visit 2 (day 0)	Visit 3 (week 1)	Visit 4 (week 2)	Visit 5 (week 3)	Visit 6 (week 4)	Visit 7 (week 8)	Visit 8 (week 12)	Visit 9 (week 24)
Informed consent	X								
Inclusion/exclusion criteria	X								
Randomization		X							
Intervention		X	X	X	X	X	X	X	
Basic information^a^	X								
Body measurement^b^	X	X	X	X	X	X	X	X	X
Vital signs	X	X	X	X	X	X	X	X	X
Medical history^c^	X								
General physical examination	X							X	X
Compliance monitoring				X					
Healthy behaviors	X							X	X
Readmission rate								X	X
Anxiety	X							X	X
Psychological capital	X							X	X
Subjective well-being	X							X	X
Clinical indicators^d^	X							X	X
Adverse event monitoring			X	X	X	X	X	X	X

^a^Age, gender, job, medical insurance, exercise, smoking habit, drinking habit, sleep pattern, etc.

^b^Height and weight, but only weight for visit 2 and follow-up

^c^Including general medical history and family history of CHD

^d^Including TG, TC, LDL, HDL, blood glucose, etc.

#### Baseline assessment

After the screening visit, if a participant fulfills the inclusion criteria and has signed the informed consent form, he will be assigned to the baseline assessment. The baseline assessment takes place at visit 1. Baseline assessment includes patients’ basic information (such as age, gender, education, income, occupation, medical insurance, comorbidity, exercise, smoking habit, drinking habit, sleep pattern, etc.), body measurement (height, body weight, body mass index (BMI), blood pressure (BP), heart rate, etc.), clinical indicators (TG, TC, LDL, and blood glucose, etc.), and questionnaires (Health Promotion Lifestyle Scale II (HPLP II), Self-rating Anxiety Scale (SAS), Positive Psychological Capital Questionnaire, General Well-Being Scale).

#### Intervention

After randomization, the patients in the control group will receive conventional nursing, and the patients in the intervention group will record positive events for 3 months in addition to conventional nursing, that is, once a week for 12 weeks. Each patient in the intervention group will be assigned a notebook to record positive events and a health education manual. The specific implementation plan is as follows.

#### Intervention group

On the basis of the same conventional nursing as the control group, the following measures were given:

1. Develop positive psychological intervention programs: the intervention plan was designed by our team based on the positive psychology intervention program by Seligman [22] and also based on a review of literature and expert consultation [27–29]. Related literature reported that the period of positive psychological intervention is generally more than 8 weeks [30]. Lan [19] conducted a 10-week intervention on type 2 diabetes patients, and their depression and anxiety psychology was also effectively improved, and their self-care ability was improved too. So, the implementation time of positive event recording in this study is planned to be 3 months, and the specific implementation methods, time, and measures are shown in Table 2.

**Table 2:** Intervention program for recording positive events in patients after PCI

Week	Intervention forms	Intervention theme	Measures
1	Face-to-face	Three positive events	Review 3 events about your positive state this week and record them (for example, I finished my exercise today, and I took medicine under the doctor’s advice today etc. It has nothing to do with the size of the matter). Once in a week
2	Online	Personal strengths	Conduct a brief assessment of your strengths, recording the strengths, as well as the practice and results. Once in a week
3	Online	Expressing gratitude	Recall the help you have received in your life, record it, and write a letter of thanks to the person. Once in a week
4	Online	Enjoyable and meaningful activities	Complete 3 enjoyable and meaningful activities on your own and keep records. Once in a week
5	Online	Experiencing success	Review previous successes, learn to enjoy them, summarize the reasons for success, and record. Three times in 1 week
6	Online	Acts of kindness	Complete three acts of kindness and record the pleasant experiences associated with them. Once in a week
7	Online	Eliminating negativity	Eliminate negativity (When there are negative emotions, record one thing that makes you happy). Three times in 1 week
8	Online	Interests and habits	Develop your own interests and hobbies, and summarize and record happy experiences related to them. Once in a week
9	Online	Accomplishing the goal	Set realistic goals and achieve them, and record the fulfilling, pleasant feelings associated with them. Once in a week
10	Online	Summarizing life	Summarize your past great life and look to the future, write down 1 − 2 pages. Once in a week
11–12	Online	Repeat training	For the next 2 weeks, repeat the above and write it down
2. Intervention implementation: using a combination of face-to-face intervention, telephone, or WeChat remote intervention. Patient recruitment and week 1 intervention instructions were completed during the patient’s hospitalization, and the researchers communicated with the patients face to face, distributed the intervention instruction manual, explained the 12-week intervention plan and matters needing attention in the exercise process, and assigned the tasks in the first week. Thereafter, the researcher will communicate with participants online (via phone or WeChat) for approximately 20 − 30 min each week. During each communication session, the researcher reviewed the patient’s recordings for the previous week, learned about the patient’s thoughts and feelings during the intervention and gave feedback, and introduced the schedule for the following week. The specific implementation is provided in Table 2.
3. Follow-up: mainly by phone or WeChat, once a week in the first month after discharge and once a month after that until 3 months. Follow-up (via phone or WeChat) will continue for 3 months after the end of the intervention, with a frequency of 3 months. Each follow-up time is about 5 − 10 min. If the patient has any questions, the time can be extended as appropriate.
4. Recording log: each individual is given a recording log and asked to record the number and time of recording every day. The data will be used to assess intervention compliance.

#### Control group

1. Conventional nursing: on the first day after enrollment, researchers communicate with patients one-on-one and explain CHD-related knowledge to patients, including risk factors, diet strategies, weight control, physical exercise, blood pressure and lipid management, etc. At the time of discharge, the charge nurse stresses the importance of medication, regular checkups, smoking cessation, exercise, and a healthy diet to the patient and distributes a health management manual for patients with coronary heart disease (The main contents of this manual include the operation process of coronary intervention, knowledge of medication, importance of review, diet management, exercise mode, and so on.).
2. Follow-up by phone or WeChat: the investigator focuses on understanding the patient’s recovery and giving health instructions. Frequency and duration of follow-up visits: once a week in the first month after discharge and once a month after that until 3 months. Follow-up will continue for 3 months after the trial at a frequency of 3 months. Each follow-up lasts about 5 − 10 min, during which patients can be consulted by phone or WeChat. It is not difficult to extend the time appropriately.

### Outcome

The primary outcome indicators are healthy behaviors, readmission rate, and anxiety level, and the secondary outcome indicators are psychological capital, subjective well-being, and clinical indicators (including TC, LDL, HDL, blood glucose, body mass index, etc.).

#### Primary outcome measure

1. Healthy behavior: The Health Promotion Lifestyle Profile II (HPLP II) will be used to measure patient’s healthy behavior. The scale was revised by American nursing scientist Walker [31], which mainly includes 6 dimensions and 52 items. The total Cronbach’s *α* coefficient of the scale is 0.94, and the Cronbach’s *α* number of each dimension is 0.79–0.87. Each item is scored by grades 1–4; the higher the score, the better the healthful lifestyle. It will be checked at visits 1, 8, and 9.
2. Readmission rate: In this study, readmission is defined as the readmission caused by cardiovascular problems, including hypertension, angina, arrhythmia, cardiac insufficiency, etc., excluding the cases of readmission due to cold, tumor, and other factors. The readmission will be followed up by telephone for 3 months. The readmission information will be collected at visits 8 and 9.
3. Anxiety: The Self-rating Anxiety Scale (SAS) will be used to measure the anxiety of patients with CHD. The scale was compiled by Chinese professor Zung (1971), there are 20 items on the scale, each item is scored by grades 1–4, and items 5, 9, 13, 17, and 19 are scored in reverse. According to the results of Chinese norm model results [32], the cut-off value of the SAS standard score is 50, of which 50–59 is mild anxiety, 60–69 is moderate anxiety, and over 69 is severe anxiety. The scale has been widely used in China and its validity and reliability have been verified (Cronbach’s *α* 0.82). The anxiety score will be measured at visits 1, 8, and 9.

#### Secondary outcome measure

1. Psychological capital: The positive Psychological Capital Questionnaire (PPQ) is used to evaluate the patient’s positive psychology. The questionnaire was revised by Professor Zhang et al. [33] of Nankai University on the positive psychological capital questionnaire compiled by Lutas et al. The questionnaire includes 26 items, Cronbach’s *α* coefficient is 0.92, and Cronbach’s *α* coefficient of each dimension is 0.81, 0.76, 0.75, and 0.78, respectively. The higher the score, the better the psychological capital. It will be measured at visits 1, 8, and 9.
2. Subjective well-being: The General Well-Being Scale (GWB) is mainly used to evaluate the subjective well-being of patients. The scale was amended by Duan [34], a Chinese scholar, on the basis of Fazio. The scale includes 18 items, and the higher the score, the stronger the subjective well-being. The internal consistency coefficient of the scale is 0.91 for males and 0.95 for females. The internal consistency coefficient of the retest is 0.85, and the correlation coefficient between the subscale and total scale is 0.56–0.88. The subjective well-being score will be measured at visits 1, 8, and 9.
3. Clinical indicators: Clinical indicators will be followed up by researchers through WeChat or telephone to collect relevant clinical laboratory indicators of patients in 3 months, including BP, blood lipid, blood glucose, BMI, etc. The clinical indicators will be collected at visits 1, 8, and 9.

#### Safety outcome measure

The safety assessment will be performed for all subjects who have been randomized and recorded positive events more than once. The subjects’ vital signs and general physical status will be examined at every visit. All participants will be required to report any adverse events (AEs) that occur during the trial of visits 2, 3, 4, 5, 6, 7, 8, and 9. All AEs that occur after the start of this trial should be recorded in the case report form, regardless of whether they are related to positive event recording or not. All AEs will be evaluated for causality.

#### Compliance monitoring and management

The researchers will collect the recording logs of the patients after 2 weeks of positive event recording for the purpose of calculating compliance. The compliance rate (%) = [continuous recorder as required/total number of interventions] × 100%. Only when the compliance is ≥ 70%, the research will continue; otherwise, find out the reasons and carry out scheme rectification.

#### Sample size calculation

Calculated according to the formula of sample size estimation based on the comparison of the mean of two independent samples [35]: *n*_1_ = *n*_2_ = 2[(*Z*_*α*_ + *Z*_*β*_)*σ*/*δ*]^2^ + *Z*^2^_α_, *σ* is the standard deviation, *δ* is the difference between the two population means, *n*_1_ and *n*_2_ are the required contents of the two samples, respectively, and *Z*_*α*_ and *Z*_*β*_ are *Z* values corresponding to the type I error probability and the type II error probability, respectively. At bilateral *α* = 0.05, power = 0.8, *Z*_*α*/2, ν_ = 1.96, *Z*_*β, ν*_ = 0.842, *σ* = 12.68, and *δ* = 10.39, the sample size for each group is 25 cases. Considering the 20% sample loss rate and other factors, the total number of samples is expected to be 80.

### Data management and quality control of data

Both the case record form (CRF) and the web-based electronic database will be used to manage individual participant data. To protect confidentiality, the files are stored in a secure and locked place and manner, and the subject identification and private information will be deleted from all study documents. Quality control of the data will be handled at two different levels: the investigators will be required to ensure the accuracy of the data as the first level of control when they input the records in CRF. The second level will include data monitoring and validation that will be carried out by two full-time graduate students who will not be involved in the intervention or data collection. All the data will be double-inputted into the computer using Epidata3.0 software. After finishing the data entry and dealing with the query, the database will be locked under the orders of the principal investigator, and the SPSS software will be used for data analysis. No one is allowed to view the database without authorization; otherwise, they should notify the principal investigator.

### Statistical analysis

The Kolmogorov–Smirnov test will be used to test the normal distribution of continuous variables. Continuous variables will be presented by mean ± standard deviation (SD) if they are normally distributed or by median with interquartile range if they are not normally distributed. For the comparison of measurement data between the two groups, a *t*-test of two independent samples will be used for normal distribution data, and the Mann–Whitney *U* rank sum test will be used for non-normal distribution data. The comparison of count data between the two groups will be performed by *χ*^2^ test or Fisher’s exact test. An independent statistics expert will perform statistical analysis in a blind manner. SPSS for windows version 25.0 (SPSS Inc., Chicago, IL, USA) will be used for statistical analysis. The statistical level of significance will be set at *P* < 0.05.

#### Auditing

This study will be an interim trial review, independent of the investigator and sponsor.

#### Protocol amendments

Any possible amendments to the protocol during the course of the trial, including changes in study objectives, sample size, or study procedures, will require formal modifications to the protocol. The modification will be agreed by the project research team, approved by the Ethics Committee of Soochow University School of Medicine prior to implementation, notified to the Chinese government’s Ministry of Science and Technology, and reported to the participants as necessary.

## Discussion

At present, CHD has become the main cause of death of urban and rural residents in China [3] and is also one of the main reasons for the economic burden of chronic non-communicable diseases [2]. Its occurrence and development are closely related to unhealthy behaviors such as unreasonable diet, smoking, drinking, and so on [36]. Only relying on drugs or surgical treatment will not change the pathological progress of coronary atherosclerosis. Studies have shown that the higher incidence of ISR and MACE is associated with unhealthy behaviors [6, 7]. Therefore, it is of profound significance to change the unhealthy behaviors of patients to reduce the occurrence of cardiovascular events.

The American College of Cardiology (ACC) and the American Heart Association (AHA) issued the “2019 cardiovascular primary prevention guidelines,” which pointed out that adhering to a healthy lifestyle is an important measure to prevent cardiovascular disease [37]. Among the 15 special activities of healthy China, measures to promote a healthy lifestyle, such as reasonable diet, tobacco control, and national exercise, have been put in a prominent position. These above show the importance of healthy behaviors to the organism. Therefore, it is necessary to help patients establish healthy behaviors.

According to the “extension-construction” theory proposed by Fredrickson et al. [38], this theory believes that positive emotions can expand the thinking and action ability, and then build a series of enduring resources of individuals, so as to promote health and development. This study is based on the positive psychology of the experimental design, paying more attention to the potential positive forces of patients, helping patients to tap their own good quality, improve positive emotions, and promote positive development. Many experimental studies have confirmed that positive psychological intervention can help patients to improve anxiety, depression, and other bad psychology; improve their quality of life; improve treatment compliance; and help people quit smoking [16–18]. This study applies positive psychological intervention to patients after PCI for the first time, through systematic practice and training, to determine whether positive event records based on positive psychology will promote the establishment or maintenance of healthy behaviors, reduce the rate of readmission, improve negative emotions such as anxiety and depression, improve subjective well-being, etc., which will provide new ideas and references for the formulation of secondary prevention strategies for CHD.

Our trial has several advantages. Firstly, the purpose of our study is to explore the effects of positive event recording on healthy behaviors, readmission rate, and negative emotions such as anxiety and depression. At home and abroad, there have been studies on the clinical application of writing positive events, and our research team has previously conducted cross-sectional surveys of patient’s healthy behaviors, readmission status, and negative emotions such as anxiety and depression after PCI, which has a solid practical and theoretical foundation. Secondly, the intervention measure of this study is to record positive events by patients themselves, which can mobilize the enthusiasm of patients to actively participate in disease management. Thirdly, positive events recording discussed in this study belong to the secondary preventive measures for patients with CHD, which is also in line with the “two heart” nursing mode advocated by China now. This form of nursing intervention research is rarely reported in the field of CHD in China.

Despite the advantages of its results, our study has the following limitations. First of all, due to limited time, manpower, and funding, this study is only conducted in a hospital in Suzhou. In future trials, more patients with PCI in different levels of hospitals should be included to explore the role of positive psychology. Secondly, only one positive psychological intervention therapy is used in this study. In the future, a variety of progressive positive psychological intervention methods can be combined to test to determine a better treatment plan.

After the completion of this study, we will conduct large-sample, multi-center, and high-quality RCTs in future studies to further verify the role of positive event recording in the maintenance and promotion of health and provide a reliable evidence-based basis for the clinical promotion and application of positive event recording.

## Trial status

The study was registered with the Chinese Clinical Trial Registry (registration number: ChiCTR2000034538) on 10 July 2020. This study started to recruit volunteers on 11 July 2020 and is expected to complete the recruitment by 31 June 2021. So far, 20 volunteers have been recruited for this trial.

## Supplementary Information

**Additional file 1.** Ethical approval documentation.

**Additional file 2.** Model consent form (Chinese).

**Additional file 3.** SPIRIT checklist.

**Additional file 4.** Funding documentation (English copy).

## Data Availability

The results of the trial will be disseminated through scientific journals or presentations at scientific conferences. This dataset is available from the corresponding author upon reasonable request.

## Abbreviations

ACC: American College of Cardiology
AE: Adverse event
AHA: American Heart Association
AIDS: Acquired immune deficiency syndrome
BMI: Body mass index
BP: Blood pressure
CHD: Coronary heart disease
CRF: Case record form
GWB: General Well-Being Scale
HDL: High-density lipoprotein
HIV: Human immunodeficiency virus
HPLP: Health Promotion Lifestyle Profile
ISR: In-stent restenosis
ITT: Intention-to-treat
LDL: Low-density lipoprotein
MACE: Major adverse cardiovascular events
PCI: Percutaneous coronary intervention
PPQ: Positive Psychological Capital Questionnaire
RCT: Randomized controlled trial
SAS: Self-rating Anxiety Scale
SD: Standard deviation
TC: Total cholesterol
TG: Triglyceride
