# Supplementary figures and images for: Effect of positive event recording based on positive psychology on healthy behaviors and readmission rate of patients after PCI: a study protocol for a prospective, randomized controlled trial
Source: Trials. 2022 Dec 13;23:1013. doi: 10.1186/s13063-022-06964-9 (PMC9746175; doi:10.1186/s13063-022-06964-9)

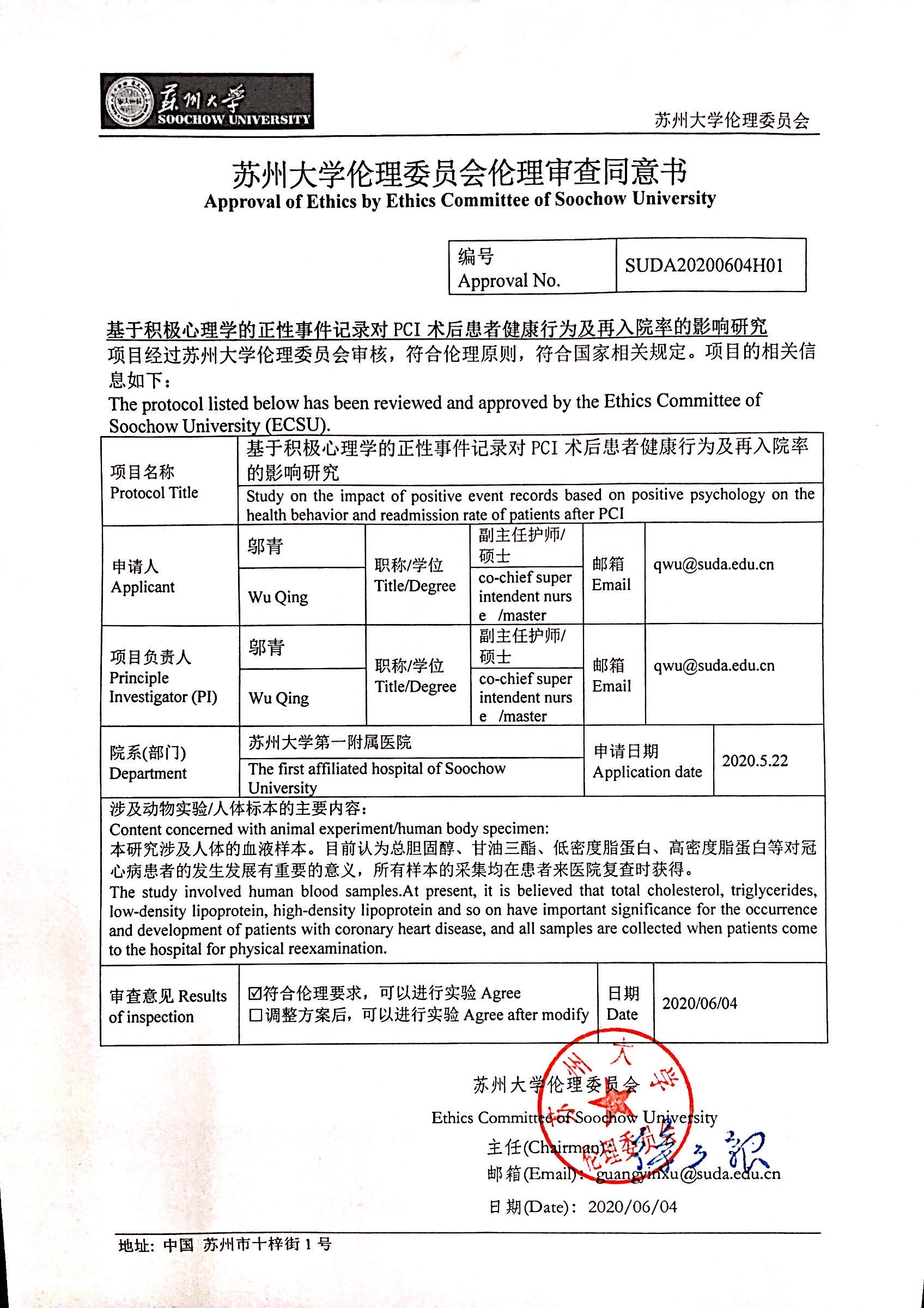

Supplement: Supplementary file 1 — Additional file 1. Ethical approval documentation. [file 13063_2022_6964_MOESM1_ESM.doc]
