# Supplementary material for: Effect of positive event recording based on positive psychology on healthy behaviors and readmission rate of patients after PCI: a study protocol for a prospective, randomized controlled trial
Source: Trials. 2022 Dec 13;23:1013. doi: 10.1186/s13063-022-06964-9 (PMC9746175; doi:10.1186/s13063-022-06964-9)
Supplement: Supplementary file 2 — Additional file 2. Model consent form (Chinese). [file 13063_2022_6964_MOESM2_ESM.doc]

**知情同意书**

尊敬的女士／先生：

我们正考虑邀请您参与一项“基于积极心理学的正性事件记录对PCI术后患者健康行为及再入院率的影响研究”的研究。在同意参与该项研究之前，您应了解此研究的具体内容，这十分重要。请认真阅读本文件，并提出问题。本项研究已通过医疗机构伦理委员会审批，是否参加本项研究完全取决于您的个人意愿。

**1．研究目的**

本研究拟通过随机对照试验的方法，在接受PCI治疗的人群中评估记录积极正性事件对健康行为和再入院率的影响，为今后冠心病患者二级预防奠定基础。

**2.背景**

冠心病（Coronary Heart Disease,CHD）是由于冠状动脉狭窄所引起的急性心肌缺血缺氧性疾病，是全球死亡率最高疾病之一^[1]^，在我国死亡率居第三位^[2]^。随着冠心病发病率和死亡率的逐年增高^[3]^，《中国卫生统计年鉴2019》显示我国居民在心绞痛方面人均花费14939.55元，在急性心肌梗死方面人均花费28879.3元。胡建平^[4]^等的研究显示我国冠心病患者医疗支出为576.89亿元，是导致慢性非传染性疾病经济负担的主要原因之一，大量的医疗费用不仅给社会增加了负担也给家庭带来沉重的打击。由此可见，冠心病的防治工作一直是全世界面对的医学难题，攻克这一医学难题也势在必行。

目前冠心病的主要治疗方法有药物治疗和血运重建治疗^[5]^（包括介入治疗和外科冠状动脉旁路移植术）等，其中经皮冠状动脉介入治疗（percutaneous coronary intervention，PCI）因创伤小、恢复快、住院时间短等特点，成为治疗冠心病的主要主要方法之一。研究显示^[6]^PCI可显著减轻冠心病症状，降低死亡率，显著改善生活质量。但仍有部分患者术后发生主要心血管不良事件（Major adverse cardiovascular events,MACE）的风险，一项研究显示，行PCI的心绞痛患者术后2d再入院率及死亡率分别为0.48%和0.02%，1个月内再入院率及死亡率分别为9.6%和0.22%^[7]^。PCI术后由于发生冠状动脉支架内再狭窄(in-stent restenosis，ISR)的概率可达5%～30%，对患者的治疗效果及预后造成不良影响，增加了术后再入院率^[8]^。

冠心病患者预后由多种因素决定，刘斌、徐琳等研究显示^[8,9]^不良行为会增加支架内再狭窄风险以及发生主要心血管不良事件的发生。另外负性心理亦可影响PCI患者术后的预后，马士容^[10]^研究了冠心病介入治疗期间心理对患者预后的影响，结果发现焦虑是影响PCI术后12个月MACE的独立影响因素；Frasure-Smith^[11]^等发现，情绪障碍与冠心病患者2年MACE发生有关；一项由曹选超^[12]^等人进行的研究发现，对老年合并心衰患者进行情绪干预，可以帮助患者改善心功能，减少跌倒事件的发生，提高生活质量；还有研究发现，对患者进行心理干预，可以提高治疗配合度，还可以帮助患者转变生活方式，建立健康行为^[13-15]^。所以，只有控制好各种已知的危险因素，才能使冠心病患者获益最大。

积极心理学^[16-17]^主要帮助人们形成良好的心理品质和行为模式，重点关注积极的主观体验、积极的人格特质和积极的社会环境，充分挖掘个体自身力量。目前积极心理学在国外已经广泛运用到妇产科、糖尿病等多个领域，Corno^[18]^等研究表明积极心理干预可以帮助孕妇减少焦虑抑郁心理、提高幸福指数；Huffman^[19]^等对2型糖尿病患者实施积极心理干预发现，可以帮助患者提高治疗依从性；Hoeppner^[20]^等研究显示积极心理干预可以帮助人们戒烟。而积极心理学在我国开展的相对较少，主要集中在教育^[21]^、精神心理疾病^[22]^等领域，蓝艳^[23]^等人的研究明确了基于积极心理的心理干预有助于帮助患者保持良好的心理状态,改善的抑郁症状,提高自护能力；朱婷^[24]^等人的研究也明确了积极心理干预能提高脑卒中住院病人的伤残接受度及自理能力。

积极心理学实施方式多种多样，有团体心理辅导^[25]^、书写表达^[26]^等，正性事件记录作为书写表达的一种，已经应用到医学领域，在减轻患者负性情绪、提高积极乐观情绪以及增加体力活动水平方面得了一定疗效^[27]^。Burton ^[28]^等人研究发现，通过书写记录正性事件可以帮助人们增进认知，Suhr ^[29]^等人的研究表明，书写正性事件对帮助精神疾病患者稳定心理健康有一定益处。国内学者袁秀娟^[30]^等研究发现，书写表达正性事件可以改善原发性肝癌患者的生活质量,尚星辰^[26]^通过给予炎症性肠病患者同样的干预，也帮助患者缓解了负性心理，增强了积极心理，促进了积极应对和提高了生活质量。由此可见，正性事件记录在帮助患者促进心理健康、提高生活质量和积极应对等方面有良好效果。

综上所述，记录正性事件可以帮助人们减轻焦虑、抑郁等不良心理，提高幸福指数，促进健康行为。故本研究拟通过随机对照实验的方法，首次在冠心病人群中评估记录正性事件对健康行为和再入院率的影响。

**3．受试者的大致数量与参与研究的预计持续时间**

预计约有60名受试者参与这项研究，根据随机数字表将符合纳入与排除标准的接受PCI治疗的患者随机分为正性事件记录干预组和对照组，干预持续**12w**。对照组采用常规护理方案，正性事件记录记录方案为在对照组的基础上，每周按照我们的要求记录一些具有积极正能量的事情。您是否参加本研究，完全取决于您的个人意愿。若您同意参加，我们将收集您的一些基本资料、临床资料情况。我们会每周进行一次电话随访或短信、QQ、微信交流，获得您对摄入正性事件记录方案的执行情况。在研究结束后（即3个月后），将再次评估、检测上述资料及相关指标；在6个月后，我们同意再次收集您的有关资料。

**4．研究过程**

本着自愿参加的原则，您可以根据自己的意愿选择参加或拒绝调查。若您同意参加，我们将按照随机数字表将您随机分为正性事件记录干预组和对照组，对照组采用常规护理方案，干预组的正性事件记录方案为在对照组的基础上，每周按照按照不同的主题进行记录，干预持续12w。并于接受干预前后收集您的基本资料、临床资料、相关指标调查问卷及相关检测指标，所有资料绝对保密，仅供研究使用。

在您了解整个研究的内容，您的问题都得到了满意的答复之后，如果您希望参加本次研究，您需签署这份知情同意书。

您可以现在提出问题，也可以在研究过程中提出问题。研究者会在研究过程中随时告知您他们所获悉的可能会改变您参与研究的决定的因素。

**5．参与研究可能的风险和不适**

本研究中除了干预提供的正性事件记录干预方案外，没有额外的干预措施。因此，参与此项研究，将不会给您增加超出常规诊疗以外的风险。在获得基本信息资料、临床实验室检查资料外无其他的风险。所有的程序均有专业的操作人员密切指导和监测，保证安全和尽可能提供舒适。另外，您的所有个人信息，我们将按相关规定严格保密。

**6．可能的获益**

您参与该项研究，我们将对您进行冠心病相关饮食、运动等指导，使您获益于相关疾病管理的知识，但您无法从中获取直接经济利益。如有新的发现，您将被告知该研究中任何可能改变您决定的信息。

**7．费用及补偿**

本研究中，受试者的记录本和签字笔费用，由研究团队提供，受试者不会得到经济报酬，但会得到健康教育手册和小礼物，作为对他们付出时间和精力的感谢。

**8．保密及隐私授权**

研究者负责遵循适用的数据保护条例来处理您的研究数据。但伦理委员会和上级行政管理部门视察能查阅到这些资料。研究结果可能会在医学刊物/会议上发表，但您的名字不会被公开。

在签署该份知情同意书后，即表明您同意研究者收集、使用和共享您的健康信息数据。在研究结束之前、研究结果得出之前，您允许我们使用您的健康信息的授权依然有效。但您可以随时通过研究负责人撤回知情同意书。

**9．自愿参与/完全或部分退出研究**

参与本次研究完全出于您个人的意愿。您可以选择不参与本项研究，您也可以在任何时候自由退出，您的任何医疗待遇与权益不会因此而受到影响，也不会受到医务人员的歧视。

**10．问题及信息**

在您签这份同意书之前，研究组所有成员都会回答您的所有问题。如果您在签署该同意书之后，依然有问题、建议或意见，您还可以跟研究者进行沟通。您可以随时了解本研究有关的信息资料和研究进展。

**研究者及电话：苏州大学医学部**[**护理学院研究生：**](mailto:护理学院研究生，林源，135-8484-1519，邮箱1024065806@qq.com)**胡尧尧，18896538393。**

**11．知情同意**

研究负责人或相关研究人员已经口头告知我本研究的有关信息，同时我也已经阅读了上述书面信息。

我得到了充分的机会就上述研究进行讨论，并提问题。我同意参加本项研究，并了解我参与该研究完全是自愿的。我了解我可以在任何时候退出研究，而且我的退出不会影响我今后的就医。签署这种份知情同意书，我同意我的个人信息数据，包括我的医疗信息数据将会按上述的方式被使用。我知道我会得到这份知情同意书的复印件。

受试者或其法人代表签字： 年 月 日

**研究者声明：**

我将严格履行科学研究道德原则和规范，履行科学家的职责，遵守法律、法规、遵守技术操作规范；树立敬业精神，遵守职业道德，尽职尽责为患者服务；关心、爱护、尊重患者，保护患者的隐私；努力钻研学术知识，提高专业技术水平；宣传卫生保健知识，对患者进行健康教育。

研究者签字： 年 月 日
