# Supplementary material for: Effect of positive event recording based on positive psychology on healthy behaviors and readmission rate of patients after PCI: a study protocol for a prospective, randomized controlled trial
Source: Trials. 2022 Dec 13;23:1013. doi: 10.1186/s13063-022-06964-9 (PMC9746175; doi:10.1186/s13063-022-06964-9)
Supplement: Supplementary file 4 — Additional file 4. Funding documentation (English copy). [file 13063_2022_6964_MOESM4_ESM.pdf]

Planning category：Minsheng science and technology project→Basic research on medical and health applications

Technology planning project contract

Project number: sys2018018

Project name: Effect of voluntary breathing exercises on stable coronary artery disease in heart rate variability and rate-pressure product

Starting and ending time: From July 1, 2018 to June 30, 2021

Project undertaking unit: The First Affiliated Hospital of Soochow University

Unit address: Box 424, 50 Donghuan Road, Suzhou city

Zip code: 215021

Project leader: Wu Qing **Phone number:** 13914007828

Finance employee in charge: Qihua Sun

Phone number: 051267500586

Project contact: Wu Qing **Phone number:** 13914007828

Suzhou science and technology bureau

January 2017

The project of "Effect of voluntary breathing exercises on stable coronary artery disease in heart rate variability and rate-pressure product " was approved by Suzhou science and technology support[2018] No.39 document as the official announcement of Suzhou city's 2018 science and technology development plan. In order to strengthen the implementation and management of science and technology planning projects, party a-Suzhou municipal bureau of science and technology, party b-Qing Wu and party c-the First Affiliated Hospital of Soochow University sign this science and technology planning project contract according to the Contract Law of the People's Republic of China.

Project leader					
Name	Gende	Age	Technical title	Department	Unit
Qing Wu	female	37	associate professor	Cardiology	The First Affiliated Hospital of Soochow University
Lead researcher					
Qi Zhao	male	37	associate professor	Radiotherapy	The First Affiliated Hospital of Soochow University
Xiaohua	female	56	associate professor	Cardiology	The First Affiliated

Wang					Hospital of Soochow University
Yunying Hou	female	37	associate professor	Cardiology	The First Affiliated Hospital of Soochow University
Lin Liu	female	35	supervisor nurse	Cardiology	The First Affiliated Hospital of Soochow University
Xin Jiang	female	32	supervisor nurse	Nursing	Wuxi People's Hospital
Zhisong He	male	38	associate professor	Cardiology	The First Affiliated Hospital of Soochow University
Yaoyao Hu	female	24	teaching assistant	School of nursing	Soochow University
Qiushi Liang	male	22	teaching assistant	School of nursing	Soochow University
Wei Zhu	female	36	associate professor	Electrocardiographic room	The First Affiliated Hospital of Soochow University
Yuan Xue	female	24	teaching assistant	School of nursing	Soochow University
Zaixiang Tang	male	37	associate professor	School of Public Health	Soochow University

[illegible]

Party a:

Legal representative of the science and technology bureau:

章

Date：06/23/2018

Party b:

Project leader:

Qing Wu

Date：06/23/2018

Party c:

Project undertaking unit:

单位科研处公章

Date：06/23/2018
